# A Successful Endovascular Treatment of Massive Hematuria Caused by Aorto-Ureteric Fistula: A Report of a Rare Case

**DOI:** 10.7759/cureus.53215

**Published:** 2024-01-30

**Authors:** Konstantinos G Seretis, Theofanis Papas, Vasileios Papaioannou, Nikolaos Giannakopoulos, Andreas M Lazaris

**Affiliations:** 1 Department of Vascular Surgery, Korgialenio-Benakio Hellenic Red Cross Hospital, Athens, GRC; 2 Department of Vascular Surgery, Attikon University Hospital, Athens, GRC

**Keywords:** aorto-ureteric fistula, medical microchip implant for humans, prolonged ureteral stenting, massive hematuria, uretero-arterial fistula

## Abstract

Fistula formation between the urinary tract and the arterial system is very rare, and usually involves the ureter and the adjacent iliac vessels. Communication of the ureter with the aorta has been described a few times worldwide, and most of them had a fatal outcome. In our case, a 79-year-old man had a history of total cystectomy for malignancy and diversion of both ureters to a single site in the right hypogastrium with the left one crossing over the aorta. He was admitted elsewhere several times for intermittent hematuria, and four months ago the diagnosis of communication of the left ureter with a mycotic aortic pseudoaneurysm was made. He was then referred to an interventional radiologist who sealed the communication. He was admitted to our hospital four months later in a state of hypovolemic shock and massive hematuria. In lack of information, it seemed to us that he had been treated with endovascular aneurysm repair (EVAR) for uretero-aortic communication, and was experiencing a regression because of endoleak formation. We attempted to treat him as type I endoleak with a proximal extension, and upon failure, with distal extensions, but finally we had to ‘build’ the entire previous graft from the inside to achieve hemodynamic stability. Our patient remained stable, without endoleak on the post-intervention computed tomography angiography (CTA). Post-operatively, we discovered that the initial operation was the formation of a bifurcated graft with multiple bare stents and coil embolization through them. This was done in an attempt to avoid material infection by the mycotic aneurysm. This is an example of a case where 'things got rough' in a lack of information on patients' medical records. Maybe the time has come to adopt the concept of implanting microchips into humans which would enable doctors to access their medical records. This will only serve as a tool for the benefit of the suffering patients, especially when we are dealing with life-threatening situations with no time to be lost.

## Introduction

Fistula formation between the urinary tract and the arterial system (UAF) is a very rare clinical condition, and usually involves the ureter and the adjacent common iliac or external iliac arteries. The first description was made in 1908 by Moschcowitz [1], and only 235 cases have been reported ever since [2]. There are scattered reports of anatomical variations in the literature, and the naming of those UAFs is made using the primary disease organ’s name first and then the targeted organ’s name. According to this nomenclature, the following groups of UAF exist: ureteroarterial fistula, aortoureteric fistula, arterio-ureteric fistula, and iliac artery-to-ureteral fistula.

Adequate diagnosis is crucial to rescue patients suffering from this life-threatening condition, and the mortality rate, which was 66% before 1980 [3], has decreased to 13% recently [4], mainly because of improvements in critical care management and advances in endovascular treatment.

## Case presentation

We present a case of fistula formation between the left ureter and the aorta at the level of the aortic bifurcation. Our patient, a 79 years old man with a history of total cystectomy for malignancy one year ago and diversion of both ureters to a single site, had both ureters catheterized with a J-stent and the left one was crossing over the aorta at the level of the aortic bifurcation. He had been admitted previously several times for intermittent hematuria and had been treated conservatively, and four months ago on his last admission for a more severe episode of hematuria, the diagnosis of aorto-ureteric fistula (AUF) had been set. He was then referred to the interventional radiology department, where they managed to seal the communication. Their initial approach was coil embolisation and deployment of bare-metal stents, choosing to avoid placement of graft material because of the risk of infection.

**Figure 1:**
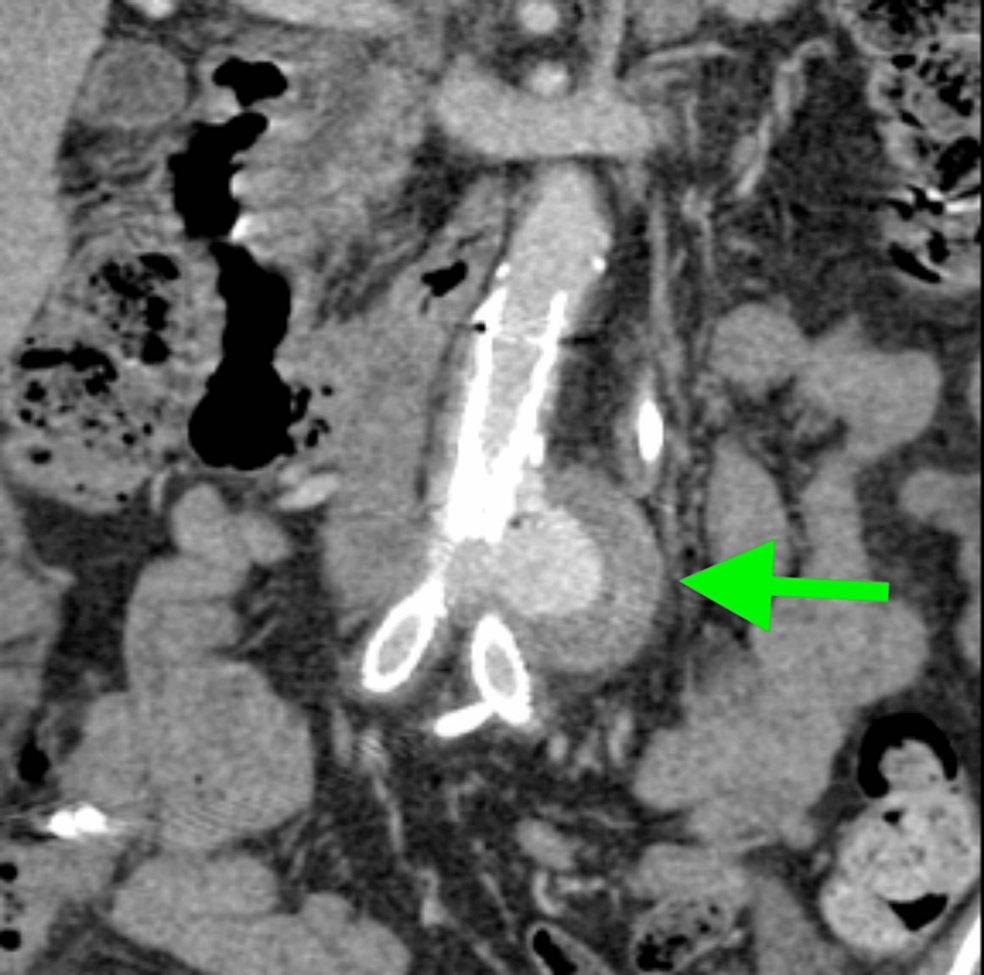
Whole body CTA on admission showing endoleak formation (green arrow). CTA: computed tomography angiography

**Figure 2:**
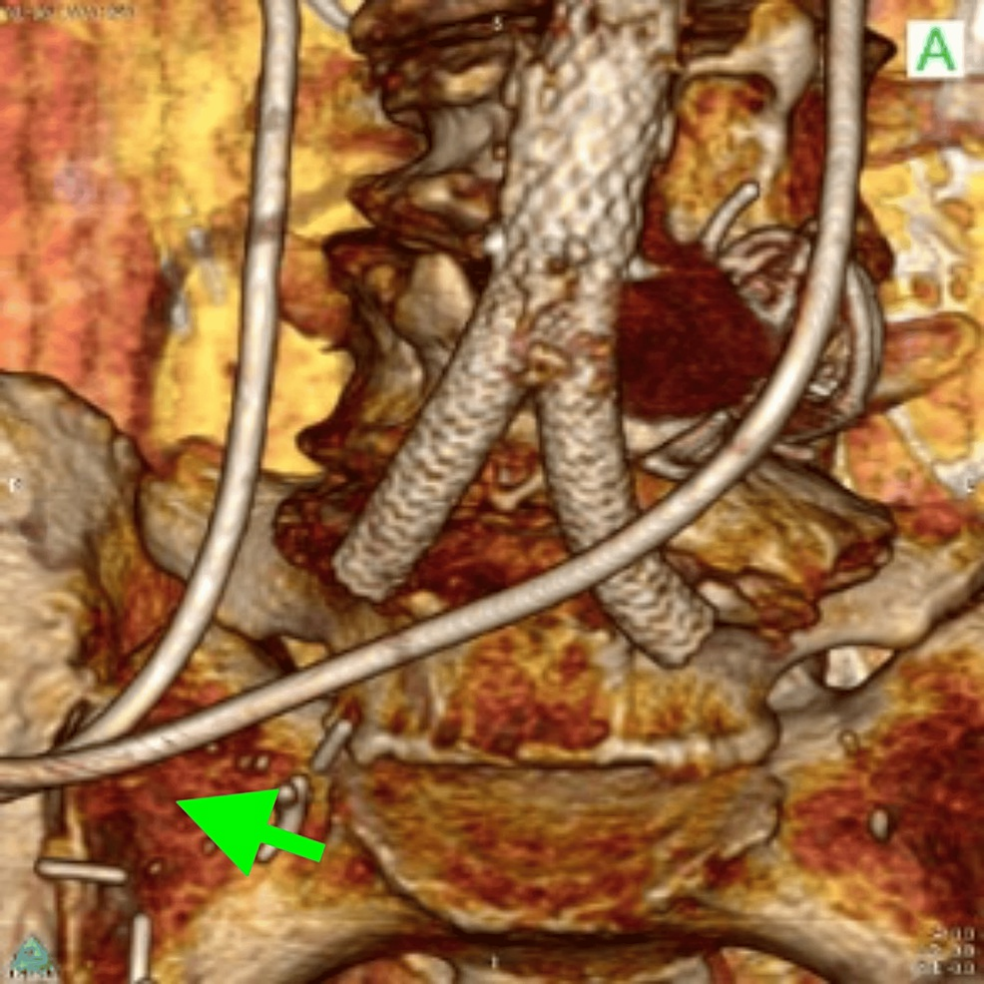
CTA on admission, 3D volume rendering, showing diversion of both ureters to a single site (green arrow). CTA: computed tomography angiography

**Figure 3:**
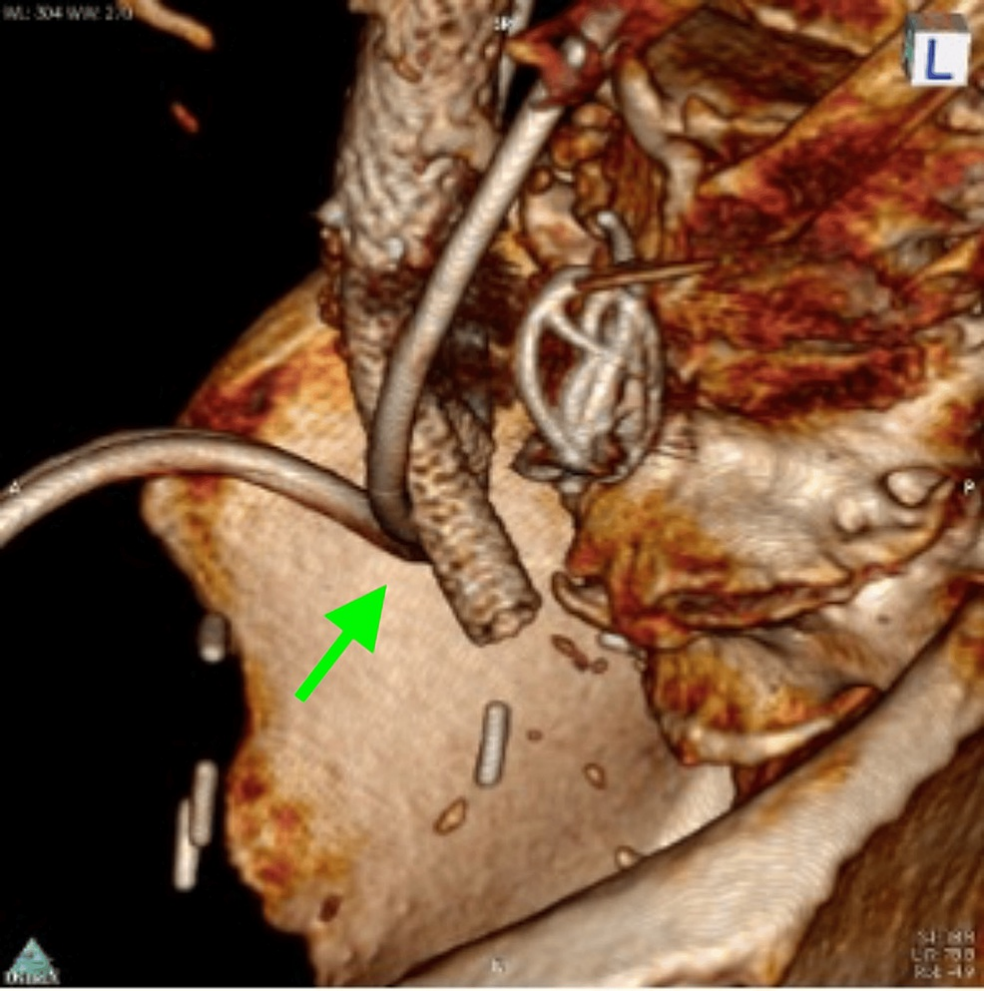
CTA on admission, 3D volume rendering, showing L ureter J stent crossing over the aortic bifurcation. CTA: computed tomography angiography

**Figure 4:**
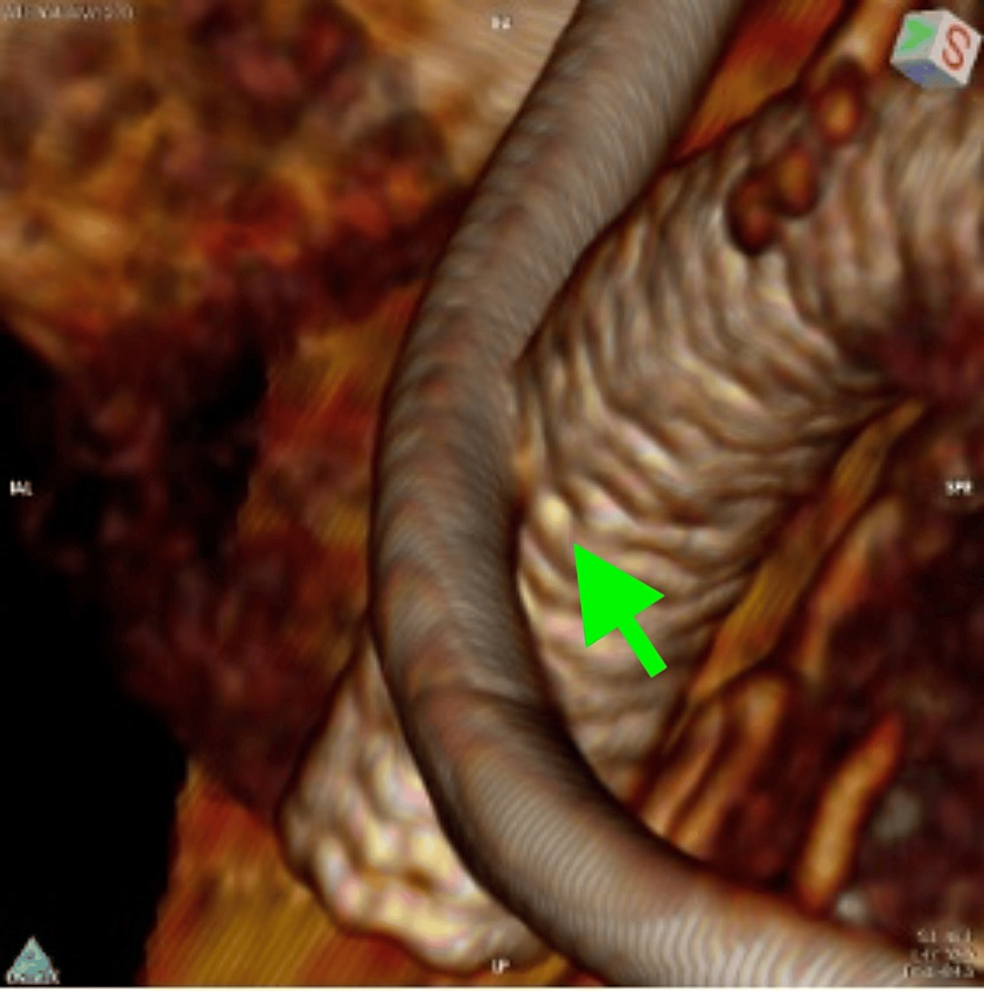
CTA on admission, 3D volume rendering, showing the site of AUF formation. CTA: computed tomography angiography, AUF: aorto-ureteric fistula

He was admitted to our hospital four months later in a state of hypovolemic shock and massive hematuria (Ht:21.4%, Hb:6,7 g/dL), with no attending person to provide information on his medical history. The diagnosis, in a lack of information on his medical records, was set after diagnostic imaging with a whole-body computed tomography angiography (CTA) scan (Figures 1-4).

**Figure 5:**
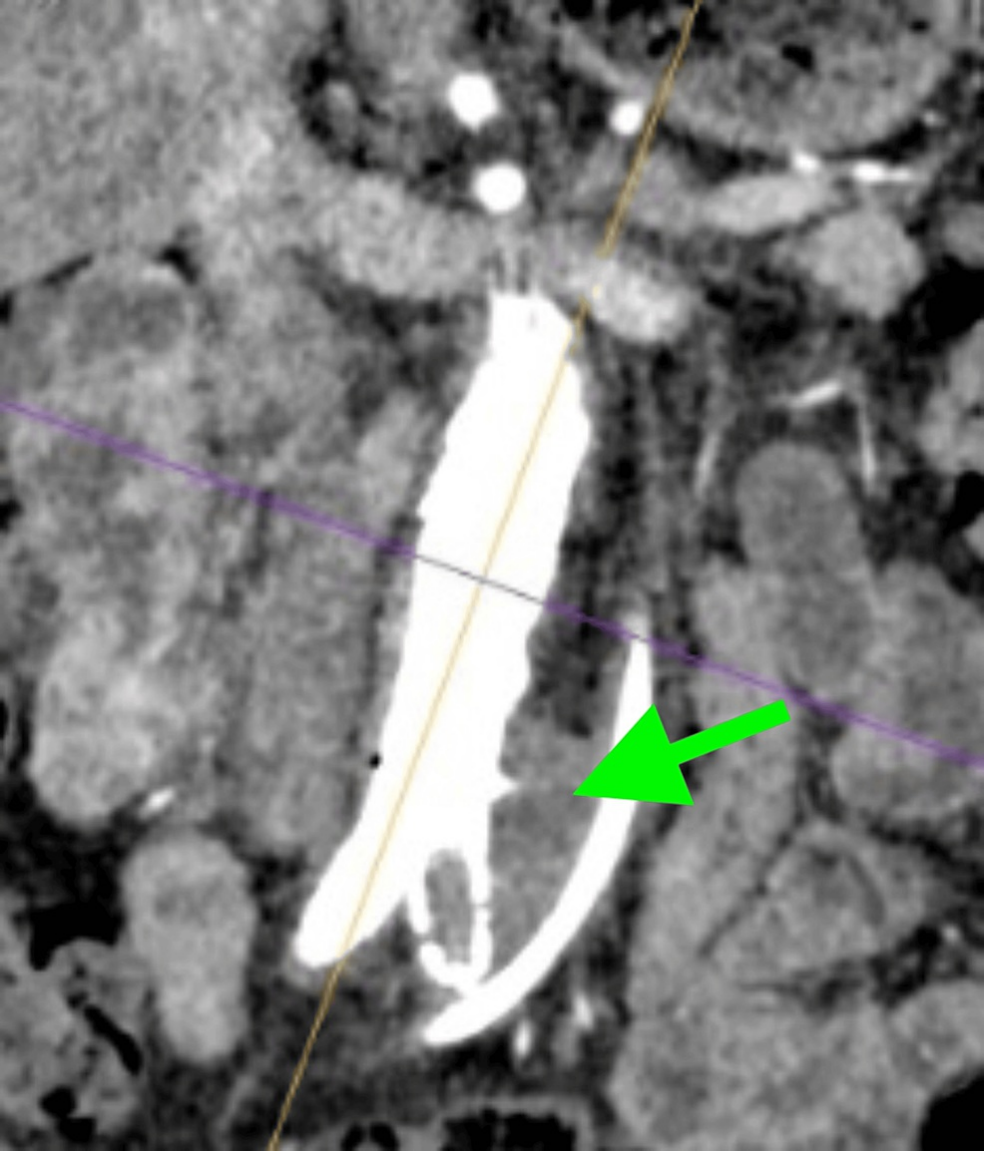
Post-procedural CTA showing control of the endoleak and no signs of extravasation in the area of AUF (green arrow). CTA: computed tomography angiography, AUF: aorto-ureteric fistula

The patient was found to have regression on his previous condition, because of endoleak formation between the ureter and the materials deployed in the previous attempt to seal the communication. Our vascular team was consulted to assist doctors in the urology department and provide emergent treatment. Without any information on his medical history, it seemed to us that he had been treated with an endovascular aneurysm repair (EVAR) for aorto-ureteric communication and now he was experiencing a regression on the basis of endoleak formation. We first attempted to treat him as a type I endoleak with a proximal extension, and upon failure, with distal extensions, but finally we had to ‘build’ the entire previous operation from the inside to achieve control of the endoleak (Figure 5).

**Figure 6:**
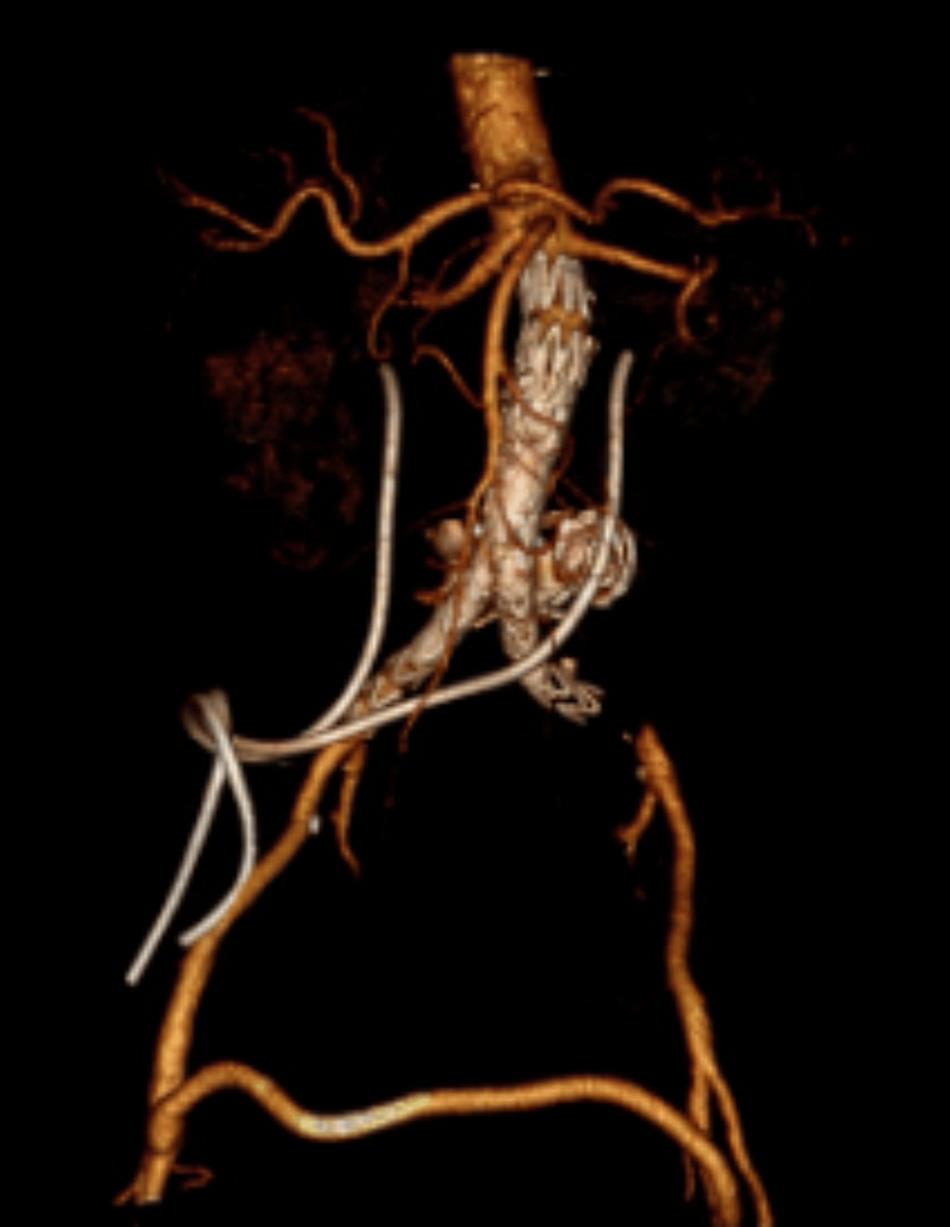
Post-procedural CTA showing the final result. CTA: computed tomography angiography

Finally, a vascular plug was placed on the left common iliac artery (LCIA) limb in an aorto-unilateral fashion, and a femoral-femoral bypass was conducted (Figure 6). This was done in an attempt to divert blood flow away from the site of communication and maximise the time to re-intervention (the graft will become contaminated eventually, and will have to be extracted in the future). The decision for this was based on the surgeon's preference and not guided by evidence, and it didn't compromise the final result.

The patient regained hemodynamic stability, and was transferred to the urologic ward. The J-stents were exchanged with new ones, and he remained hemodynamically stable during the remaining course of his treatment.

## Discussion

Although fistula formation between the urinary tract and the UAF is a rare clinical occurrence, adequate diagnosis and critical care management are crucial to rescue a patient suffering from this life-threatening condition. The vascular defect can be repaired using the open surgical approach, embolization techniques, ligation and extra-anatomic arterial reconstruction, or endovascular stenting [5]. The introduction of endovascular management to treat UAF [6] has been advocated for the reduction observed in mortality recently, although it has to be stressed out that every treatment option has its pros and cons, and decision-making should be individualized for every case.

Risk factors for UAF formation include history of pelvic surgery, previous radiation therapy, previous vascular surgery in the region and history of ureteral stenting [7]. Most of the cases presented in the modern era are linked with prolonged ureteral stenting, and the number of known cases has notably increased since the introduction of ureteral stents in 1978 [8] from 12 reported cases until that time to 235 cases reported in the most recent review that was published in 2022 [2]. Repeated arterial pulsations over a rigid material that can lead to subsequent necrosis of the ureter and the arterial wall have been described as the causative mechanism of fistula formation.

It is important to mention that patients who have been treated with vascular prosthetic materials, because of the complexity and the expensive nature that these materials have, should carry on them (in a similar manner to pacemakers) an informative tab for future reference. Maybe it's time to adopt the concept of implanting microchips into humans which would enable doctors to access their medical records. It has been almost 20 years since the first FDA-approved microchip implant for humans was introduced [9], but fears that it could lead to a violation of patients' privacy have hindered its widespread adoption. In our case, if we had access to our patient’s medical records and knew what was the initial approach, we would have treated him with an easier and safer method. We would have treated him with EVAR, instead of ‘building’ one from covered stents, something that resulted in a greater risk of endoleak formation in the future. The consolation to this is that this operation, although lifesaving, only serves as a ‘bridge’ to the definite treatment. The implanted endograft has to be removed in the near future because of the increased risk of infection of the graft material, a complication that has to be avoided because of its catastrophic nature [10].

## Conclusions

UAF is a rare cause of massive hematuria, and only early diagnosis and treatment can improve the prognosis of this life-threatening condition. Risk factors for fistula formation include prior surgery or radiation in the pelvic region and prolonged ureteral stenting. The number of reported cases has increased dramatically since the introduction of ureteral stents, but the reported mortality has been reduced. Endovascular management to treat UAF is responsible for the observed reduction in mortality and has been the treatment of choice lately, although it carries an acceptable risk of endograft infection. Our case highlights the importance of instant access to the medical records of a patient suffering from an emergent, life-threatening condition, and supports the idea of implanting microchips into humans, with the aim to enable doctors to easily access their medical records.
